# Alzheimer's Disease Analysis Algorithm Based on No-threshold Recurrence Plot Convolution Network

**DOI:** 10.3389/fnagi.2022.888577

**Published:** 2022-05-10

**Authors:** Xuemei Li, Tao Zhou, Shi Qiu

**Affiliations:** ^1^School of Mechanical and Electrical Engineering, Chengdu University of Technology, Chengdu, China; ^2^School of Computer Science and Engineering, North Minzu University, Yinchuan, China; ^3^Key Laboratory of Spectral Imaging Technology CAS, Xi'an Institute of Optics and Precision Mechanics, Chinese Academy of Sciences, Xi'an, China

**Keywords:** Alzheimer's disease, EEG, PLV, recursive graph, no-threshold

## Abstract

Alzheimer's disease is a neurological disorder characterized by progressive cognitive dysfunction and behavioral impairment that occurs in old. Early diagnosis and treatment of Alzheimer's disease is great significance. Electroencephalography (EEG) signals can be used to detect Alzheimer's disease due to its non-invasive advantage. To solve the problem of insufficient analysis by single-channel EEG signal, we analyze the relationship between multiple channels and build PLV framework. To solve the problem of insufficient representation of 1D signal, a threshold-free recursive plot convolution network was constructed to realize 2D representation. To solve the problem of insufficient EEG signal characterization, a fusion algorithm of clinical features and imaging features was proposed to detect Alzheimer's disease. Experimental results show that the algorithm has good performance and robustness.

## Introduction

Alzheimer's disease is a degenerative disease of the central nervous system, mainly manifested as progressive memory impairment, cognitive dysfunction, personality change and language impairment, and other neuropsychiatric symptoms, which seriously affect social, career, and life functions. Alzheimer's disease is a common disease in the elderly, and its prevalence and incidence are extremely high. According to statistics, the incidence of Alzheimer's disease is 5%, the disease is the most common type of dementia in the elderly, accounting for 50–70% of Alzheimer's disease, common in people over 65 years old. It is of great significance to study it.

### Alzheimer's Disease

Alzheimer's disease occurred in elderly and senile prophase, characterized by progressive cognitive dysfunction and behavioral impairment of nervous system diseases, main show is memory disorders, aphasia, disuse, agnosia, visual spatial ability damage, abstract thinking and calculation ability damage, personality and behavior change, and so on, can be improved by drug treatment, and the disease is not cured. The etiology and pathogenesis of Alzheimer's disease are extremely complex, and may be related to genetic factors, brain pathological changes and other factors. Generally, Alzheimer's disease tends to occur in people over 65 years old. Mental stimulation, trauma, neurological diseases and other factors can induce Alzheimer's disease. The main pathological changes were amyloid precursor protein gene on chromosome 21, *PSEN1* gene on chromosome 14, and *PSEN2* gene mutation on chromosome 1. The brain was reduced in size and weight, and the typical histopathological changes were neuroinflammatory plaques, neurofibrillary tangles, and neuron loss (Yoon et al., 2022). Alzheimer's disease is usually silent onset, pre-dementia, and dementia stage symptoms are different, but generally manifested as memory impairment, speech loss or emotional apathy, crying and laughing impermanent, severe patients can be complicated with lung infection, urinary tract infection and pressure ulcers, and other diseases. Early diagnosis and early treatment is of great significance.

### Method

The current examination methods mainly include: neuropsychological test, hematological examination, neuroimaging examination, Electroencephalography (EEG), cerebrospinal fluid testing, genetic testing. Due to the convenience of EEG collection, it has a good detection effect for early Alzheimer's disease to become the main research direction. To this end, we used EEG for the study. Morabito et al. (2012) constructs the model analysis of Alzheimer's disease EEG from the perspective of energy entropy. Anh et al. (2012) used support vector machine (SVM) to cluster EEG. Falk et al. (2012) analyzed the disease by the variability in EEG amplitude. Hulbert and Adeli (2013) combine EEG and imaging information to make a diagnosis of the disease. Morabito et al. (2013) proposed the EEG enhancement algorithm to highlight the area where the lesion signal is located. Zhao and He (2014) used a deep learning network for disease diagnosis. Cassani et al. (2014) extracted useful information from the EEG to conduct the research on Alzheimer's disease. Bhat et al. (2015) combined the clinical neural data and EEG to conduct the study. Al-Jumeily et al. (2015) was diagnosed by EEG analysis. Al-Nuaimi et al. (2016) analyzed EEG from the perspective of amplitude to diagnose early Alzheimer's disease. Yu et al. (2016) analyzed EEG, the signal transmission process. Kulkarni and Bairagi (2017) used SVM to extract the significant features of the EEG signal. Deng et al. (2017) constructed a multiscale model from an entropy perspective to analyze the complex EEG. Chikara et al. (2018) proposed monetary reward and punishment to response inhibition modulate activation and synchronization within the inhibitory brain network. Houmani et al. (2018) built multiple networks to implement disease analysis. Kim and Kim (2018) analyzed the correlation between the signals and extracted the features. Yang et al. (2018) studied the multi-channel data of EEG and proposed parallel revolutionary recurrent neural network to realize Alzheimer's disease recognition. Chen et al. (2020) constructed a model from the perspective of classification to realize signal analysis. Yu et al. (2019) introduced the fuzzy learning theory to analyze the EEG signals. Maturana-Candelas et al. (2019) constructed a multiscale model to extract EEG features. Chikara and Ko (2019) used hierarchical model to neural activities classification of human inhibitory control, which achieved good results. Rossini et al. (2020) proposed markers for early Alzheimer's disease diagnosis, demonstrating the validity of the EEG analysis. Qiu et al. (2020) analyzed the EEG transmission process. Oltu et al. (2021) proposed a novel Alzheimer's disease detection algorithm based on EEG. Li et al. (2021) analyzed the correlation between multiple channels to diagnose the disease. Puri et al. (2022) proposed the Kolmogorov Complexity diagnosis of Alzheimer's disease. Ding et al. (2022) proposed the Alzheimer's disease automatic detection system based on EEG.

In conclusion, the diagnosis of Alzheimer's disease based on EEG has achieved some results. However, there are still the following problems in computer processing: (1) The correlation between different channels is not studied. (2) The EEG signal is not well visualized and difficult to analyze. (3) With limited characteristics and insufficient characterization.

In view of the difficult problem of analysis of Alzheimer's disease, we use computer to assist diagnosis. (1) Analyze the corresponding relationship between different channels at the same time and build PLV network structure. (2) One-dimensional EEG signals are converted into two-dimensional recurrence plot to achieve visual analysis of features. (3) Combining clinical features with EEG signals features to realize diagnosis of Alzheimer's disease.

## Algorithm Framework

**Figure 1:**
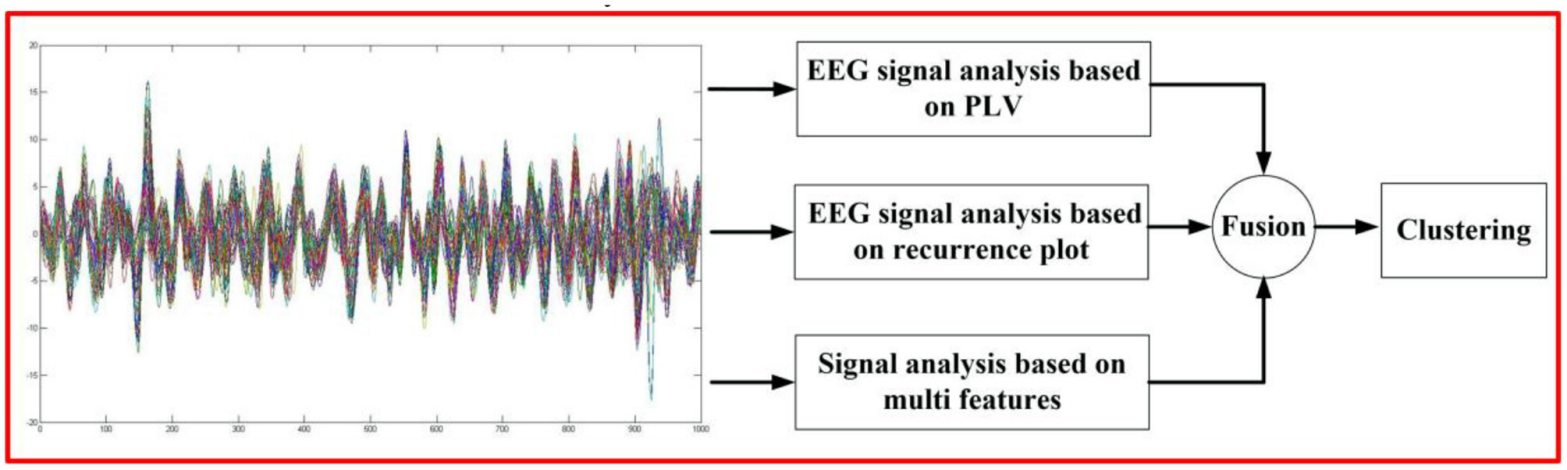
The proposed algorithm flow chart.

Through the analysis of EEG signals, we constructed a new Alzheimer's disease analysis algorithm, and the block diagram is shown in Figure 1. The model is constructed from the perspective of cognition, and the EEG signal analysis model based on Phase Locking Value (PLV) is proposed to simulate the EEG transmission process. From the correlation of EEG time series, the EEG signal analysis algorithm based on recurrence plot is proposed to convert one-dimensional information into two-dimensional information for intuitive analysis. From the perspective of feature correlation, multi-source features are extracted in order to build a model, and finally realize the fusion of decision sets and Alzheimer's disease analysis.

### EEG Signal Analysis Based on PLV

Research shows that the cognitive process of human brain designs the activities of various brain regions and the information dissemination and interaction between different functional regions (Sarma and Barma, 2022). From the perspective of computer, this process can be regarded as building a network between relevant brain regions to reflect the relationship between mutual transmissions and processing. Since EEG signal has phase synchronization relationship, we use PLV to measure EEG phase synchronization relationship:


(1) $$PLV=\frac{1}{N}\left|\sum_{j=0}^{N-1}\text{exp}\left(i\Delta \varphi \left(t\right)\right)\right|$$

(2) $$\begin{array}{rcl} \Delta \varphi \left(t\right) & = & \varphi_{x}\left(j\Delta t\right)-\varphi_{y}\left(j\Delta t\right) \end{array}$$

Where, ϕ_*x*_(*t*) and ϕ_*y*_(*t*) represent the instantaneous phase of *x*(*t*) and *y*(*t*), respectively, Δϕ(*t*) represents the phase difference, Δ*t* represents the period of application. Clustering coefficient can measure the degree of brain function separation, and the proportion of the number of connections and the maximum number of connections between a node and adjacent nodes.

The clustering coefficient of node *i* is defined as:


(3) $$\begin{array}{r} C_{i}=\frac{\underset{k\neq i}{\sum }\underset{l\neq i,l\neq k}{\sum }c_{ik}c_{il}c_{kl}}{\underset{k\neq i}{\sum }\underset{l\neq i,l\neq k}{\sum }c_{ik}c_{il}} \end{array}$$

where *c*_*ij*_ is the weight between nodes *i* and *j* of the adjacency matrix. The characteristic path length L represents the minimum number of edges of two nodes connected in the network.

The weighted network is expressed as:


(4) $$\begin{array}{r} L=\frac{N\left(N-1\right)}{\overset{N}{\underset{i=1}{\sum }}\overset{N}{\underset{j\neq i}{\sum }}\left(1/L_{ij}\right)} \end{array}$$

where *N* represents the number of weighted nodes and *L*_*ij*_ represents the number of edges of the shortest path of nodes *i* and *j*.


(5) $$\begin{array}{r} G=\frac{1}{N\left(N-1\right)}\overset{N}{\underset{i=1}{\sum }}\overset{N}{\underset{j\neq i}{\sum }}L_{ij}^{-1} \end{array}$$

Local subnet efficiency is


(6) $$\begin{array}{r} L_{ei}=\frac{1}{N_{G_{i}}\left(N_{G_{i}}-1\right)}\underset{i,k\in G_{i}}{\sum }L_{{}_{j,k}}^{-1} \end{array}$$

where *N*_*G*_*i*__ is the number of nodes of the subgraph *G*_*i*_. The centrality of the network is introduced for measurement:


(7) $$\begin{array}{r} b_{i}=\underset{m\neq i,\neq n}{\sum }\frac{\sigma_{mn}\left(i\right)}{\sigma_{mn}} \end{array}$$

**Figure 2:**
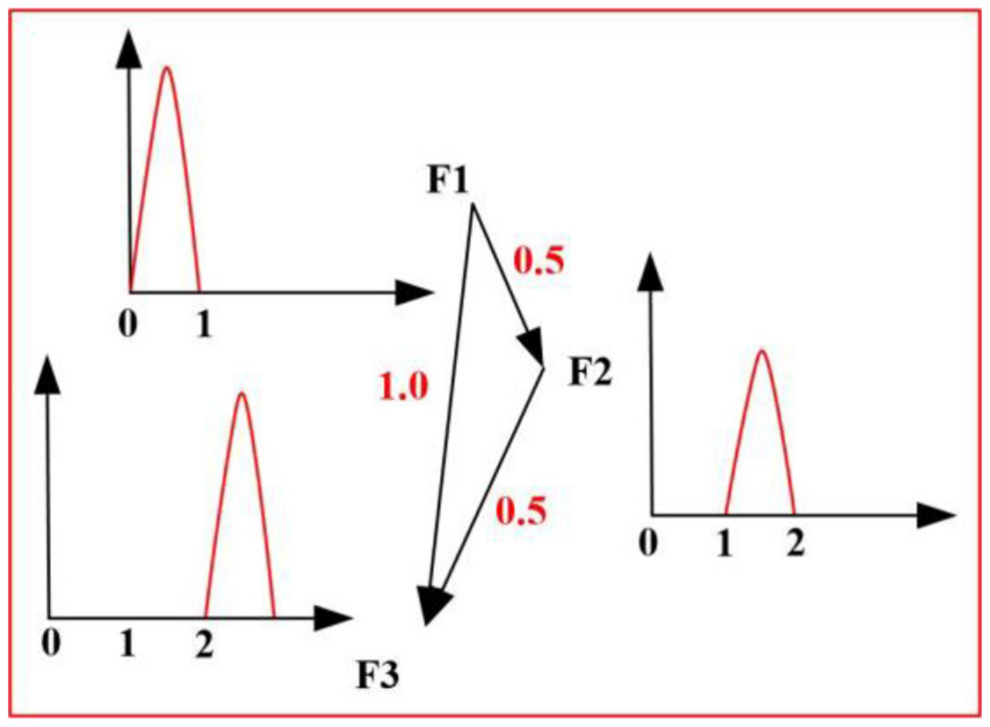
The calculation process.

Where, σ_*mn*_(*i*) represents the number of shortest paths from node m to node *n*, which goes through *i*. σ_*mn*_ represents the shortest path length from *m* to *n*. As shown in Figure 2, the signal starts F1 and ends F3 through two branches. We take *i* =2 and $b_{2}=\frac{0.5+0.5}{0.5+0.5+1.0}=\frac{1}{2}$ to achieve the centrality measure.

Under the condition of network establishment, it is necessary to extract features from the signal as input. Common Space Pattern (CSP) is used to extract airspace information. It is an efficient spatial filtering algorithm whose goal is to create an optimal common spatial filter (Kumar et al., 2017). We use CSP to extract features. CSP obtains the most distinguishing feature vector by diagonalizing the task covariance matrix. The specific process is as follows:

Given two types of data samples *X*_1_ and *X*_2_, the corresponding covariance matrix is


(8) $$\begin{array}{r} R_{i}=\frac{X_{i}X_{i}^{T}}{trace\left(X_{i}X_{i}^{T}\right)} \end{array}$$

The corresponding mixed space covariance matrix is


(9) $$\begin{array}{r} R_{c}={\bar{R}}_{1}+{\bar{R}}_{2} \end{array}$$

Where, ${\bar{R}}_{1}$ and ${\bar{R}}_{2}$ represent the average covariance matrix of two types of tasks.

Principal component analysis is applied to decompose eigenvalues of *R*_*c*_:


(10) $$\begin{array}{r} R_{c}=U_{c}\Lambda_{c}U_{c}^{T} \end{array}$$

Where, *U*_*c*_ represents eigenvector matrix and Λ_*c*_ represents eigenvalue. The corresponding whitening matrix is


(11) $$\begin{array}{r} P=\frac{U_{c}^{T}}{\sqrt{\Lambda_{c}}} \end{array}$$

The spatial filter *P* is constructed to meet the following conditions:


(12) $$\{\begin{array}{l} S_{1}=PR_{1}P^{T}=B\Lambda_{1}B^{T}\\ S_{1}=PR_{2}P^{T}=B\Lambda_{2}B^{T}\\ \Lambda_{1}+\Lambda_{2}=I \end{array}$$

Calculate the projection matrix, and whiten the transformation of the eigenvector corresponding to the maximum eigenvalue in EEG and max (Λ_1_, Λ_2_) to achieve the best classification. To do so, a projection matrix is built:


(13) $$\begin{array}{r} W={\left(B^{T}P\right)}^{T} \end{array}$$

EEG data characteristics are obtained:


(14) $$\begin{array}{r} Z_{M\times N}=W_{M\times M}*X_{M\times N} \end{array}$$

Select the maximum values of 2*m* row from *Z*_*M*×*N*_ as feature input, which is input into the constructed PLV network to realize feature classification.

### EEG Signal Analysis Algorithm Based on Recurrence Plot

**Figure 3:**
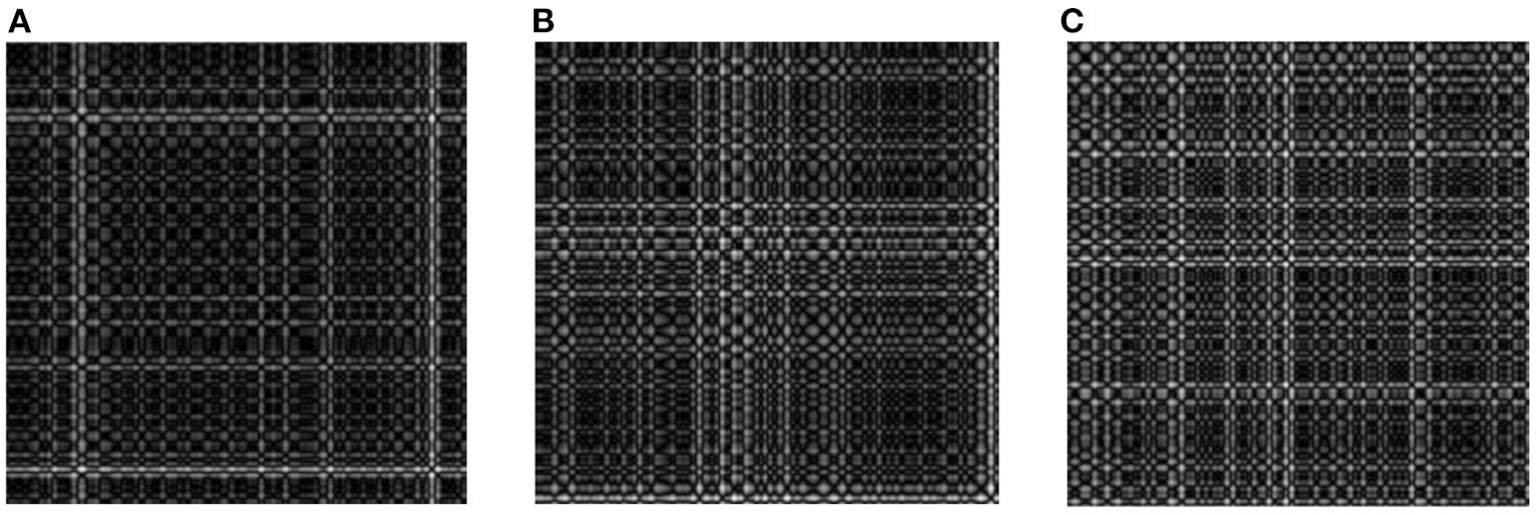
Alzheimer's disease recurrence plot. **(A)** Calm. **(B)** Morbidity. **(C)** Transitional period.

Recurrence plot can be used to measure the correlation of time series. Its core idea is to map the trajectory of moving state to the plane, which can realize visualization as shown in Figure 3. The set of time series is marked as *X*, and the corresponding recursion diagram is:


(15) $$\left\{ \begin{array}{l} R_{ij}=\varphi \left(\varepsilon -r_{ij}\right)\;i,j\in \left\{1,2,....,N-\left(m-1\right)\tau \right\}\\ r_{ij}=\left\|X\left(i\right)-X\left(j\right)\right\|\\ \varphi =\;\left\{ \begin{array}{l} 1\;x\geq 0\\ 0\;other \end{array}\right. \end{array}\right.$$

According to the recursive state of two times, *i* and j represent the horizontal and vertical coordinates of the image, and the matrix *R* composed of 0 and 1 is obtained.

Although the recurrence plot can intuitively express the time series, it increases the threshold ϕ. The richer nonlinear dynamic characteristics are lost and the characterization is incomplete. Thus, we improve it as follows to retain its characteristics to the greatest extent:


(16) $$\begin{array}{r} ER_{ij}={{\left|\varepsilon -r_{ij}\right|}_{}{{}_{}}_{}}_{}i,j\in \left\{1,2,....,N-\left(m-1\right)\tau \right\} \end{array}$$

Convolutional neural network (CNN) network has shown unique advantages in target segmentation and recognition, and has the invariance of translation, scaling and tilt of network structure. It is usually composed of input layer, convolution layer, pooling layer, full connection layer, and output layer.

**Figure 4:**
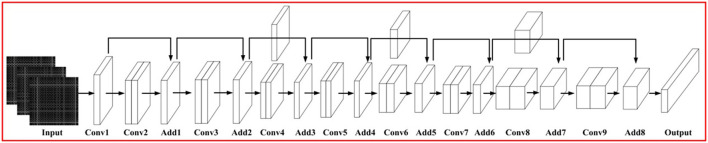
Network structure.

With the increase of network layers, the network has nonlinear fitting ability and improves the performance of the model. But it will also be accompanied by the phenomenon of gradient disappearance. In order to solve this problem, we introduce the residual block to construct the relationship between input and output through fitting the residual mapping of multi-layer networks is shown in Figure 4. The problem of difficult convergence of the deep-seated network can be solved according to certain overlapping rules. The structure is shown in Table 1.

**Table 1:** Network parameters.

**The network layer**	**Parameters**
Conv 1	(7, 7, 64); D = 2
Conv 2	(3, 3, 64) × 2; Maxpooling; D = 2
Conv 3	(3, 3, 64) × 2; Maxpooling; D = 2
Conv 4	(3, 3, 128) × 2
Conv 5	(3, 3, 128) × 2
Conv 6	(3, 3, 256) × 2
Conv 7	(3, 3, 256) × 2
Conv 8	(3, 3, 512) × 2
Conv 9	(3, 3, 128) × 2

Based on the above introduction, PLV was used to analyze the correlation between signals and calculate the probability PE of signal attributes. To obtain the probability RE of signal attributes, a network based on non-threshold recursive plot was built from the time correlation of EEG signals. We collected the age, gender, basic diseases (hypertension, hyperlipidemia, diabetes), eye movement test, etc., and selected the patients with statistically significant characteristics using *p* < 0.05. Age, diabetes, and eye movement tests were significant by screening.

## Experiment and Result Analysis

There are two data, (1) http://adni.loni.usc.edu/; (2) Clinical data collected by the hospital. The frequency of signal acquisition is 8–30 Hz, 62 channels of data. With the consent of the patients, 100 patients with Alzheimer's disease at different stages were collected including 48 women and 52 men aged 55–80 years. The EEG collected was divided into calm, morbidity, and transitional period according to professional physicians and clinical manifestations. Total 1,000 points of data were collected in each period. Construct data sets and conduct experiments.

### Introduction of Experimental Parameters and Evaluation Indexes

We analyzed the characteristics of EEG signals and sampled the data. For each EEG signal accord to the principle of average sampling, we obtained 1,000 data points, and formed the recursive plot data of 1,000 × 1,000 data. Then, subsequent experiments were conducted on this basis. In order to ensure the consistency of the experiment, we preprocessed the EEG signal data. Through data analysis, to ensure the consistency of the experiment, EEG signal data were preprocessed and representative Fp1, Fp2, F3, and F4 were normalized.

Accuracy *A* is used to measure the performance of different algorithms:


(17) $$\begin{array}{r} A=\frac{TP+TN}{TP+FP+TN+FN} \end{array}$$

Where, *TP* is the positive sample with correct model classification, *FP* is the negative sample with wrong model classification, *TN* is the negative sample with correct model classification, and *FN* is the positive sample with wrong model classification.

### Performance of PLV Algorithm

**Figure 5:**
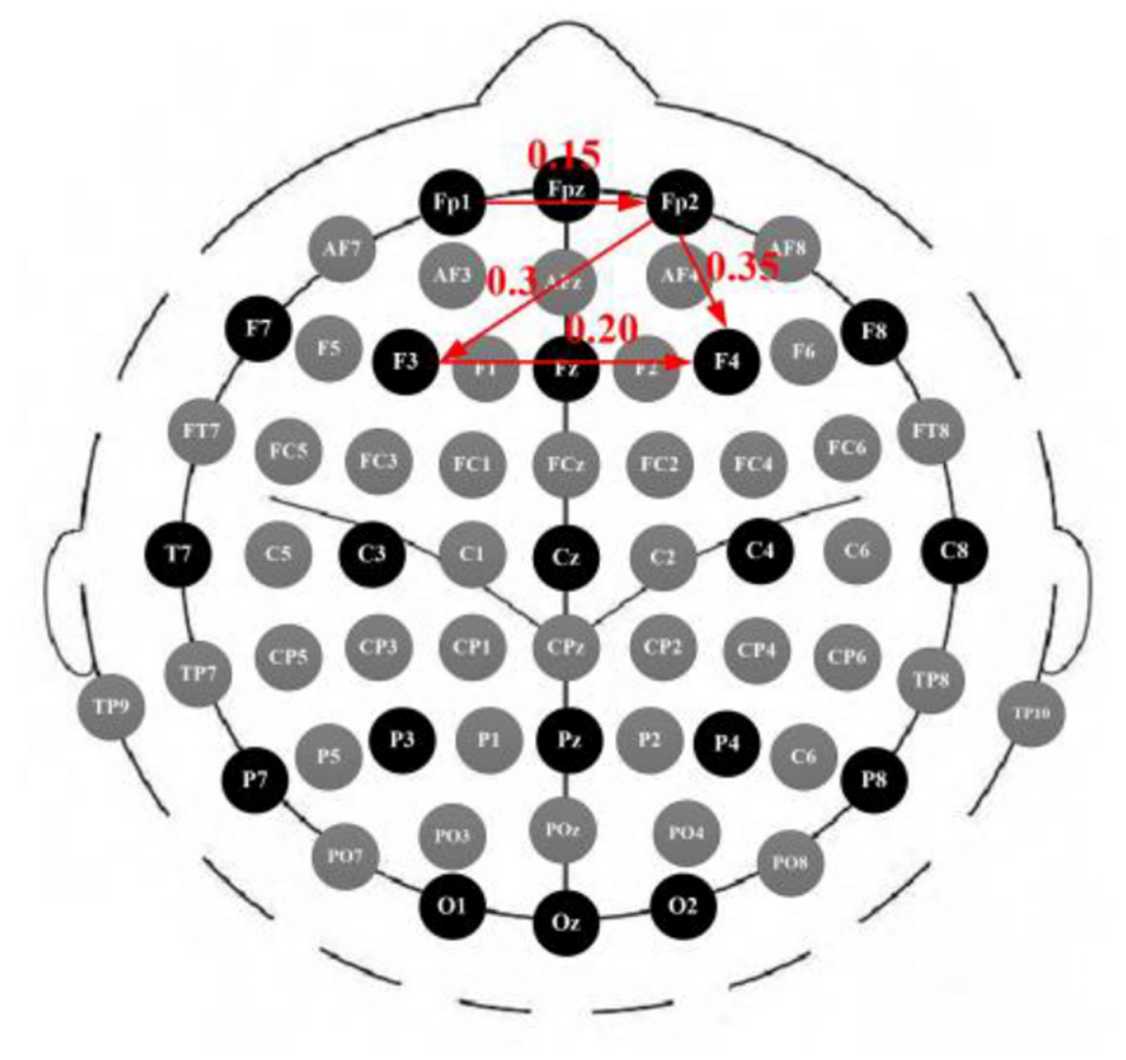
EEG PLV.

We build the brain network graph *G* = (*V, E*) and using EEG click as network nodes. The graph side shows the channel relationship. The PLV can be used as a synchronicity measure to represent the connection strength in a weighted network analysis. The results for Alzheimer's disease are shown in Figure 5, with a low degree of connection in Fp1. The connection degree between Fp2 and F3 and F4 is high, so the study is carried out based on this.

### EEG Signal Analysis of Recurrence Plot

**Figure 6:**
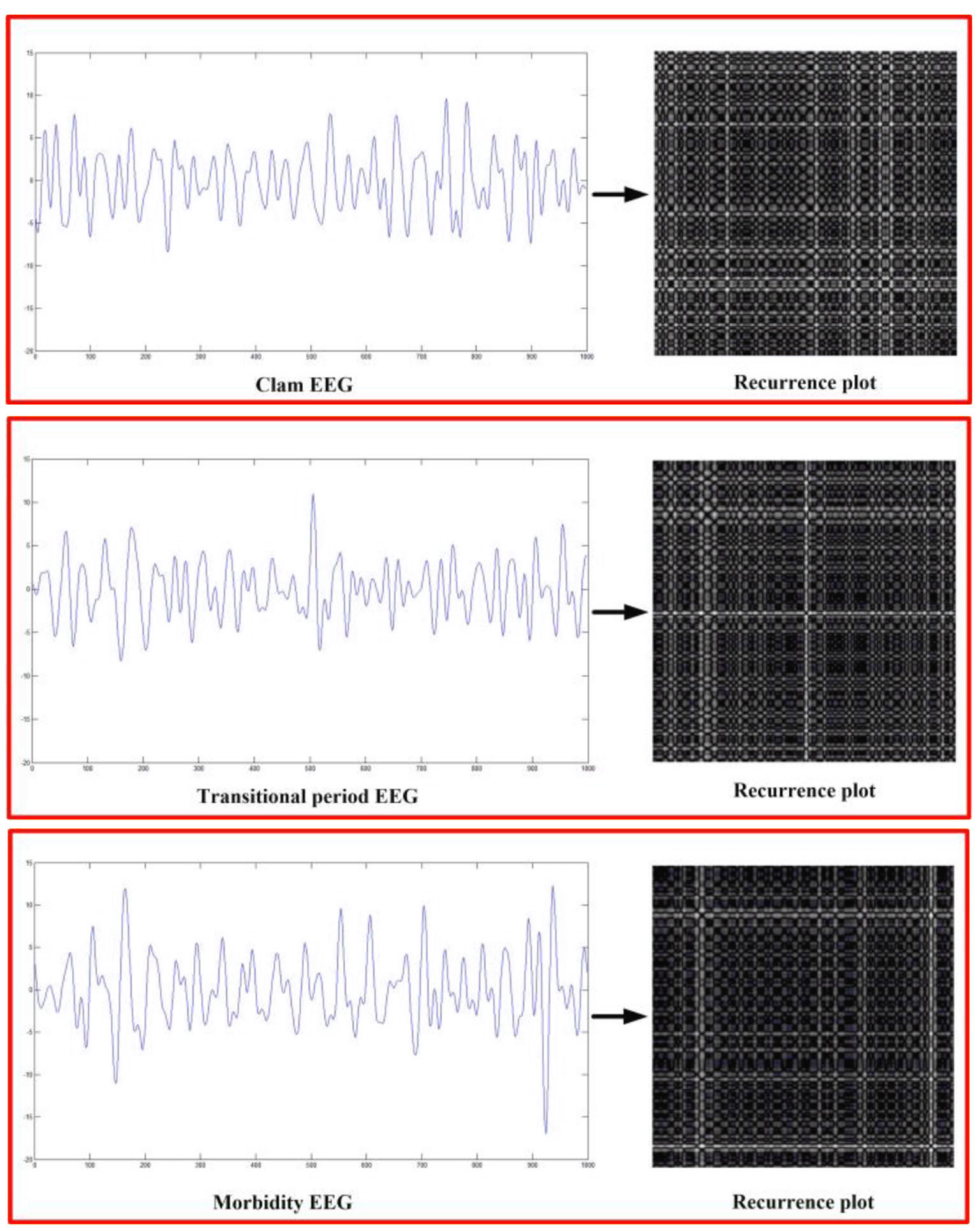
Recurrence plot.

We explored the recurrence plot by selecting EEG during periods of calm, transition and onset as shown in Figure 6. From the analysis of EEG signal, during the calm period, EEG does not fluctuate much, and the signal in the lower right corner of the recursion graph is strong. In transition, the EEG considerably began from smooth band, in the middle of the recursive plot chart presents signal is stronger. During the onset of the disease, the EEG amplitude was further enlarged, but it was not obvious on the EEG alone and could not be distinguished effectively, and the signal intensity around the recursion diagram was strong. Based on this, the three can be distinguished. Subsequent fusion of PLV and clinical features can further improve the detection effect.

**Figure 7:**
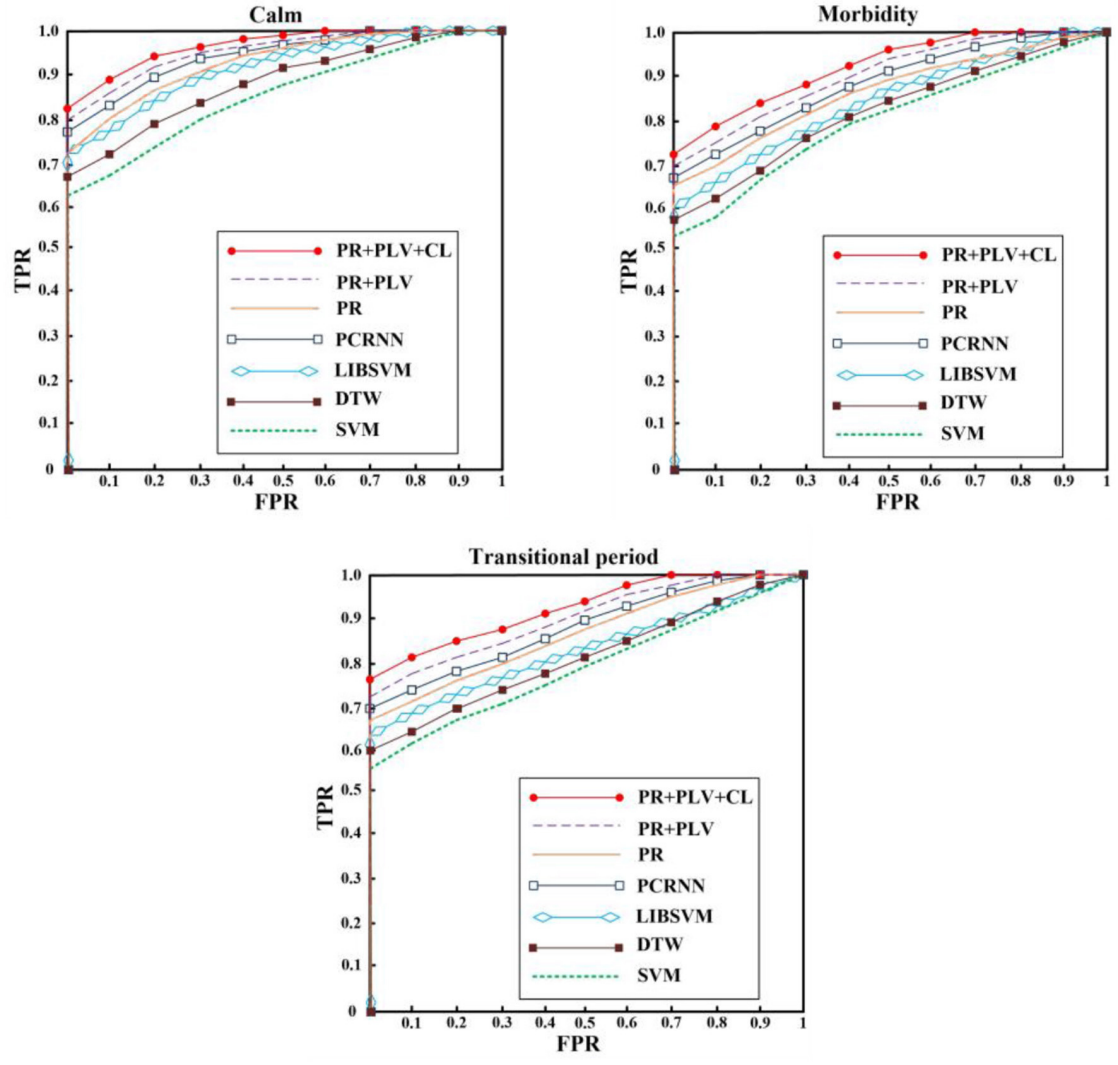
Alzheimer's disease ROC curve.

The ROC curve corresponding to our algorithm is compared with the mainstream algorithm, as shown in Figure 7. SVM algorithm (Anh et al., 2012) constructed the classifier and realized the classification of Alzheimer's disease. Parallel revolutionary Cyclic Neural Network (PCRNN) (Yang et al., 2018) established depth model and analyzed signal composition. Libsvm classifier (Chen et al., 2020) constructs the model from the perspective of classification to realize signal analysis. DTW can realize the measurement of time series. The idea of DTW is to extend and shorten two time series to represent signal similarity with the shortest distance. However, EEG signals have a strong correlation, and the number of points collected by the EEG signal is too large, which will lead to information loss and error information introduction through DTW extension and shortening. Result in poor effect. Figure 7 quiet period for acquisition of data, due to the quiet period EEG signals is relatively stable, our algorithm and comparison algorithm can better on the test. Figure 7 shows the data collected in the transitional period. The EEG gradually fluctuates from a relatively stable signal. However, due to the limited amplitude of fluctuation, the detection effect of the algorithm decreases compared with that in the calm period. Figure 7 shows the data collected during the onset of the disease, and the EEG fluctuates greatly, which can be detected by changing the amplitude. Overall, all algorithms performed best for quiet Alzheimer's disease, followed by morbidity and transitional Alzheimer's disease. In addition, the algorithm establishes a model from the perspective of EEG, carries out processing, recurrence plot and auxiliary features of EEG Alzheimer's disease, and realizes EEG Alzheimer's disease analysis, with high performance.

We added comparative experiments, and the PR algorithm proposed transformed 1D features into 2D PR without threshold, which achieved certain results in the diagnosis of Alzheimer's disease. On this basis, PLV was adopted to analyze the correlation between different channels at the same time, and the detection effect was further improved. Finally, we simulate the process of physician diagnosis, and fuse the clinical features into the model to achieve the best effect.

## Conclusions and Discussions

Alzheimer's disease is a central nervous system variable disease, although there is no effective treatment method, but it has a positive effect on its early diagnosis and early treatment. Studies show that EEG has non-invasive and easy acquisition characteristics, which has proved to be an effective means to detect Alzheimer's disease, for which we propose a new Alzheimer's disease analysis algorithm.

Early AI algorithms conducted the analysis only from a single signal perspective, ignoring the response relationship between different channels at the same time, resulting in the limited representational ability of the established model. With the improvement of medical and information acquisition ability, scholars focus their attention to the signal transmission process to build a model, which enhances the model representation ability. After deeply studying the EEG transmission process, the PLV model is constructed to simulate the EEG transmission process to obtain the Alzheimer's disease transmission characteristics.

EEG can be regarded as a time series signal, and the traditional algorithm only builds the model from the 1-dimension perspective to carry out the study of similarity measures. Due to the complexity of the signal, a unified 1-dimensional model cannot be constructed. In this paper, 1-dimensional EEG is transformed into 2D recurrence plot to measure signal similarity in an intuitive way, and construct a threshold-free mechanism to quantify similarity. On this basis, a deep-learning network is constructed to simulate the cognitive process of physicians and obtain Alzheimer's disease signal characteristics.

A large number of clinical data show that Alzheimer's disease is very closely related to clinical characterization, and modeling from the signaling perspective alone has certain limitations. Clinical data collected from patients show that people with hypertension and diabetes have a high probability and rapid progression of Alzheimer's disease.

In this paper, based on EEG signals, signal transmission, signal similarity, and clinical characterization are combined to achieve the detection of Alzheimer's disease. Experiments show that the algorithm has strong robustness and detection rate. Subsequently, we will continue to collect data to expand the data set and carry out annotation and feature mining of typical data to assist doctors in accurate diagnosis.

## Data Availability Statement

The original contributions presented in the study are included in the article/supplementary material, further inquiries can be directed to the corresponding authors.

## Author Contributions

All authors made significant contributions to the manuscript.

## Funding

This work is supported by Natural Science Foundation of China under Grant No. 62062003, Key Research and Development Project of Ningxia (Special projects for talents) under Grant No. 2020BEB04022, and North Minzu University Research Project of Talent Introduction under Grant No. 2020KYQD08. Science and Technology Rising Star of Shaanxi Youth under Grant No. 2021KJXX-61.

## Conflict of Interest

The authors declare that the research was conducted in the absence of any commercial or financial relationships that could be construed as a potential conflict of interest.

## Publisher's Note

All claims expressed in this article are solely those of the authors and do not necessarily represent those of their affiliated organizations, or those of the publisher, the editors and the reviewers. Any product that may be evaluated in this article, or claim that may be made by its manufacturer, is not guaranteed or endorsed by the publisher.

## References

[B1] Al-Jumeily D. Iram S. Vialatte F. B. Fergus P. Hussain A. (2015). A novel method of early diagnosis of Alzheimer's disease based on EEG signals. Sci. World J. 2015, 931387. 10.1155/2015/931387 25688379 PMC4320850

[B2] Al-Nuaimi A. H. Jammeh E. Sun L. Ifeachor E. (2016). “Changes in the EEG amplitude as a biomarker for early detection of Alzheimer's disease.” in 2016 38th Annual International Conference of the IEEE Engineering in Medicine and Biology Society (EMBC) (Orlando, FL: IEEE). 993–996. 10.1109/EMBC.2016.7590869 28268491

[B3] Anh V. H. Van M. N. Ha B. B. Quyet T. H. (2012). “A real-time model based support vector machine for emotion recognition through EEG.” in 2012 International Conference on Control, Automation and Information Sciences (ICCAIS) (Saigon, Vietnam: IEEE). 191–196. 10.1109/ICCAIS.2012.6466585

[B4] Bhat S. Acharya U. R. Dadmehr N. Adeli H. (2015). Clinical neurophysiological and automated EEG-based diagnosis of the alzheimer's disease. Eur. Neurol. 74, 202–210. 10.1159/000441447 26588015

[B5] Cassani R. Falk T. H. Fraga F. J. Kanda P. A. Anghinah R. (2014). The effects of automated artifact removal algorithms on electroencephalography-based Alzheimer's disease diagnosis. Front. Aging Neurosci. 6, 55. 10.3389/fnagi.2014.00055 24723886 PMC3971195

[B6] Chen T. Ju S. Ren F. Fan M. Gu Y. (2020). EEG emotion recognition model based on the LIBSVM classifier. Measurement. 164, 108047. 10.1016/j.measurement.2020.108047

[B7] Chikara R. K. Chang E. C. Lu Y. C. Lin D. S. Lin C. T. Ko L. W. (2018). Monetary reward and punishment to response inhibition modulate activation and synchronization within the inhibitory brain network. Front. Hum. Neurosci. 12, 27. 10.3389/fnhum.2018.00027 29545745 PMC5837970

[B8] Chikara R. K. Ko L. W. (2019). Neural activities classification of human inhibitory control using hierarchical model. Sensors. 19, 3791. 10.3390/s19173791 31480570 PMC6749522

[B9] Deng B. Cai L. Li S. Wang R. Yu H. Chen Y.. (2017). Multivariate multi-scale weighted permutation entropy analysis of EEG complexity for Alzheimer's disease. Cogn. Neurodyn. 11, 217–231. 10.1007/s11571-016-9418-9 28559952 PMC5430241

[B10] Ding Y. Chu Y. Liu M. Ling Z. Wang S. Li X.. (2022). Fully automated discrimination of Alzheimer's disease using resting-state electroencephalography signals. Quant. Imaging. Med. Surg. 12, 1063. 10.21037/qims-21-430 35111605 PMC8739099

[B11] Falk T. H. Fraga F. J. Trambaiolli L. Anghinah R. (2012). EEG amplitude modulation analysis for semi-automated diagnosis of Alzheimer's disease. EURASIP J. Adv. Signal Process. 2012, 1–9. 10.1186/1687-6180-2012-19224723886

[B12] Houmani N. Vialatte F. Gallego-Jutgl,à E. Dreyfus G. Nguyen-Michel V. H. Mariani J.. (2018). Diagnosis of Alzheimer's disease with Electroencephalography in a differential framework. PloS ONE. 13, e0193607. 10.1371/journal.pone.0193607 29558517 PMC5860733

[B13] Hulbert S. Adeli H. (2013). EEG/MEG-and imaging-based diagnosis of Alzheimer's disease. Rev Neurosci. 24, 563–576. 10.1515/revneuro-2013-0042 24259242

[B14] Kim D. Kim K. (2018). “Detection of early stage Alzheimer's disease using EEG relative power with deep neural network.” in 2018 40th Annual International Conference of the IEEE Engineering in Medicine and Biology Society (EMBC) (Honolulu, HI: IEEE). 352–355. 10.1109/EMBC.2018.8512231 30440409

[B15] Kulkarni N. N. Bairagi V. K. (2017). Extracting salient features for EEG-based diagnosis of Alzheimer's disease using support vector machine classifier. IETE J. Res. 63, 11–22. 10.1080/03772063.2016.1241164

[B16] Kumar S. Mamun K. Sharma A. (2017). CSP-TSM: optimizing the performance of Riemannian tangent space mapping using common spatial pattern for MI-BCI. Comput. Biol. Med. 91, 231–242. 10.1016/j.compbiomed.2017.10.025 29100117

[B17] Li K. Wang J. Li S. Yu H. Zhu L. Liu J.. (2021). Feature extraction and identification of Alzheimer's disease based on latent factor of multi-channel EEG. IEEE Trans. Neural Syst. Rehabilitation Eng. 29, 1557–1567. 10.1109/TNSRE.2021.3101240 34329166

[B18] Maturana-Candelas A. Gómez C. Poza J. Pinto N. Hornero R. (2019). EEG characterization of the Alzheimer's disease continuum by means of multiscale entropies. Entropy. 21, 544. 10.3390/e21060544 33267258 PMC7515033

[B19] Morabito F. C. Labate D. Bramanti A. La Foresta F. Morabito G. Palamara I.. (2013). Enhanced compressibility of eeg signal in alzheimer's disease patients. IEEE Sens. J. 13, 3255–3262. 10.1109/JSEN.2013.2263794

[B20] Morabito F. C. Labate D. La Foresta F. Bramanti A. Morabito G. Palamara I. (2012). Multivariate multi-scale permutation entropy for complexity analysis of Alzheimer's disease EEG. Entropy. 14, 1186–1202. 10.3390/e1407118628559952

[B21] Oltu B. Akşahin M. F. Kibaroglu S. (2021). A novel electroencephalography based approach for Alzheimer's disease and mild cognitive impairment detection. Biomed Signal Process Control. 63, 102223. 10.1016/j.bspc.2020.102223

[B22] Puri D. Nalbalwar S. Nandgaonkar A. Wagh A. (2022). “EEG-Based Diagnosis of Alzheimer's Disease Using Kolmogorov Complexity.” *in Applied Information Processing Systems*. Springer, Singapore. 157–165. 10.1007/978-981-16-2008-9_15

[B23] Qiu S. Li J. Cong M. Wu C. Qin Y. Liang T. (2020). Detection of solitary pulmonary nodules based on brain-computer interface. Comput. Math. Methods Med. 2020, 4930972. 10.1155/2020/4930972 32617117 PMC7312740

[B24] Rossini P. M. Di Iorio R. Vecchio F. Anfossi M. Babiloni C. Bozzali M.. (2020). Early diagnosis of Alzheimer's disease: the role of biomarkers including advanced EEG signal analysis. Report from the IFCN-sponsored panel of experts. Clin Neurophysiol. 131, 1287–1310. 10.1016/j.clinph.2020.03.003 32302946

[B25] Sarma P. Barma S. (2022). Emotion Recognition by Discriminating EEG Segments With High Affective Content From Automatically Selected Relevant Channels. IEEE Trans. Instrum. Meas. 71, 4000812. 10.1109/TIM.2022.3147876

[B26] Yang Y. Wu Q. Qiu M. Wang Y. Chen X. (2018). “Emotion recognition from multi-channel EEG through parallel convolutional recurrent neural network.” *in 2018 International Joint Conference on Neural Networks (IJCNN)* (Rio de Janeiro: IEEE). 1–7. 10.1109/IJCNN.2018.8489331

[B27] Yoon S. Kim S. E. Ko Y. Jeong G. H. Lee K. H. Lee J.. (2022). Differential expression of MicroRNAs in Alzheimer's disease: a systematic review and meta-analysis. Mol. Psychiatr. 1–9. 10.1038/s41380-022-01534-6 35264731

[B28] Yu H. Lei X. Song Z. Liu C. Wang J. (2019). Supervised network-based fuzzy learning of EEG signals for Alzheimer's disease identification. IEEE Trans Fuzzy Syst. 28, 60–71. 10.1109/TFUZZ.2019.2903753

[B29] Yu M. Gouw A. A. Hillebrand A. Tijms B. M. Stam C. J. van Straaten E. C.. (2016). Different functional connectivity and network topology in behavioral variant of frontotemporal dementia and Alzheimer's disease: an EEG study. Neurobiol. Aging. 42, 150–162. 10.1016/j.neurobiolaging.2016.03.018 27143432

[B30] Zhao Y. He L. (2014). “Deep learning in the EEG diagnosis of Alzheimer's disease,” in Asian Conference on Computer Vision. Springer, Cham. 340–353. 10.1007/978-3-319-16628-5_25

